# Long‐term outcomes of outpatient laser ablation for recurrent non‐muscle invasive bladder cancer: A retrospective cohort study

**DOI:** 10.1002/bco2.120

**Published:** 2021-10-13

**Authors:** Sarika Grover, Siddarth Raj, Beth Russell, Elsie Mensah, Rajesh Nair, Ramesh Thurairaja, Muhammad Shamim Khan, Kay Thomas, Sachin Malde

**Affiliations:** ^1^ Faculty of Life Sciences and Medicine King's College London London UK; ^2^ Translational Oncology and Urology Research King's College London London UK; ^3^ Department of Urology Guy's and St Thomas' NHS Foundation Trust London UK

**Keywords:** bladder cancer, laser, outpatient, progression, recurrence

## Abstract

**Objectives:**

The objective of this study is to determine the long‐term efficacy and safety of office‐based Holmium:YAG laser ablation for the treatment of recurrent non‐muscle‐invasive bladder cancer (NMIBC).

**Methods:**

We retrospectively reviewed the medical records of all consecutive patients who underwent office‐based laser ablation for recurrent bladder cancer between 2008 and 2016. The following data were collected: original histology, date of original histology, date of laser ablation, number of repeat laser ablation procedures required, date of tumor recurrence or progression, number of general anesthesia procedures (transurethral resection or cystodiathermy) required after first laser ablation, and number and severity of complications. Kaplan–Meier survival curves were produced for recurrence‐free survival, progression‐free survival, and overall survival.

**Results:**

A total of 97 patients, with an average age of 84 (62–98) years and an average Charlson Comorbidity Index of 6.9 (4–13), were included. The median follow‐up was 61 (2–150) months. Fifty‐five (56.7%) patients presented with tumor recurrence, and the median recurrence‐free survival time was 1.69 years (95% CI 1.20–2.25). Only 9 (9.3%) patients had evidence of tumor progression to a higher grade or stage, 8 (89%) of which initially had low‐grade tumors; however, no patient progressed to muscle‐invasive disease. The median progression‐free survival time was 5.70 years (95% CI 4.10–7.60), and the median overall survival time was 7.60 years (95% CI 4.90–8.70). No patient required emergency inpatient admission after laser ablation.

**Conclusion:**

Office‐based Holmium:YAG laser ablation offers a safe and effective alternative method for treating low‐volume, low‐grade recurrent NMIBC, especially in elderly patients with significant co‐morbidity, while avoiding general anesthesia and inpatient admission.

## 1 INTRODUCTION

Non‐muscle‐invasive bladder cancer (NMIBC) is the most prevalent form of bladder cancer, accounting for 70–75% of cases at presentation.^1^ According to the European Association of Urology (EAU) guidelines, NMIBC can be stratified into low, intermediate, or high‐risk depending on grade, stage, tumor size, number of tumors, presence of carcinoma‐in‐situ (CIS), and prior recurrence rate.^1^ While high‐grade tumors have a high risk of progression to muscle‐invasive disease, patients with low‐grade tumors are at extremely low risk of progression or cancer‐specific mortality.^2^, ^3^ Surveillance and treatment protocols are therefore based upon this stratification, with a less intense approach for low‐risk tumors.

Low‐grade tumors account for approximately 50% of NIMBC, and although the rate of progression and cancer‐specific mortality is low, the long‐term rate of recurrence is relatively high at 46–62%, especially if large or multifocal.^4^ The aim of managing low‐grade tumors is to therefore minimize recurrence rates, prolong the time to recurrence, reduce patient inconvenience, and minimize healthcare costs, without compromising oncological control.^5^ The most common approach for treating recurrent NMIBC is transurethral resection of the bladder tumor (TURBT) or cysto‐diathermy under general or regional anesthesia. However, less burdensome and less invasive options have been recommended for the management of low‐grade recurrences, including office‐based laser ablation or diathermy and surveillance.^6^, ^7^ TURBT carries risks of perioperative morbidity and mortality, especially in the elderly and comorbid population of patients with bladder cancer. Furthermore, repeat TURBT procedures result in considerable healthcare costs; hence, bladder cancer is currently the most expensive cancer to treat, from diagnosis to death.^8^ Office‐based laser ablation aims to treat recurrences and therefore reduce the need for repeated inpatient procedures. This technique has been shown to reduce the cost of managing NMIBC by almost a third when compared to TURBT in the operating room without compromising oncological control in the short term.^9^

However, the long‐term efficacy of laser ablation for recurrent NMIBC is not widely reported. This study aims to report the long‐term effectiveness of office‐based laser ablation of recurrent NMIBC in terms of recurrence rate, time to recurrence, and progression.

## 2 MATERIAL AND METHODS

This is a retrospective study of all consecutive patients who underwent office‐based laser ablation with a flexible cystoscope for NMIBC at a single center between 2008 and 2016, focusing on long‐term follow‐up. The following patients were included: patients with recurrent NMIBC (solitary or multiple, tumor size <2 cm) who had a primary histology of Ta‐T1 and G1‐G3 and patients over the age of 60 years. All patients in the present series were high risk for general anesthesia (GA). The exclusion criteria were diagnosis of muscle‐invasive bladder cancer (MIBC), large (>2 cm) tumors at time of recurrence, and those with primary CIS. Electronic records were searched and the following data collected: age, original histology, date of original histology, previous intravesical therapies (bacillus Calmette‐Guerin, BCG, and mitomycin C, MMC), date of recurrences, number of recurrences, histology of recurrences, any tumor progression, date of laser ablation, number of repeat laser ablations, number of TURBT procedures required after the first laser ablation, number of emergency inpatient admissions within 30 days of first laser ablation, and subsequent cystectomy, radiotherapy, or chemotherapy. Histopathological grade was reported using both 1973 and 2004 WHO classification systems. Recurrence was defined as a tumor that recurred at any point after initial laser ablation. Progression was defined as a tumor that developed into a higher grade or stage, relative to the original histology recorded, at any point after initial laser ablation on subsequent bladder biopsy or transurethral resection. The Charlson Comorbidity Index was reported for each patient, and this was used to calculate an estimated 10‐year survival score for patients in this cohort.^10^

### 2.1 Procedure

All patients were treated in an outpatient setting with a 30 W Holmium:YAG laser. A single‐dose of intramuscular gentamicin was given 30 min prior to the procedure; no analgesia was required pre‐ or post‐procedure. The procedure itself was carried out in an aseptic manner. Prior to cystoscopy, 11 ml of Instillagel® (Farco‐Pharma GmbH, Cologne, Germany) was administered. Thereafter, a 16.5‐F flexible video cystoscope was used to assess the location of all tumors. Enhanced cystoscopic techniques (e.g., narrow band imaging and blue light cystoscopy) were not used. A Holmium:YAG laser was then used to ablate any recurrent tumors, with a 365‐ or 200‐nm fiber at 0.6–0.8Js energy and rates of 10–15 Hz. Normal saline solution was used as irrigation fluid. Biopsies of papillary recurrences were not usually taken, but in those that had suspicious flat lesions, a biopsy and urine cytology were taken. In those with a history of CIS undergoing surveillance, urine cytology was taken in addition to the surveillance cystoscopy. Patients were asked to void before discharge. No patients were given immediate intravesical therapy following the procedure. Complications were assessed immediately after the procedure and then through notes review at 30 days. All patients underwent initial follow‐up 3 months after laser ablation, with subsequent cystoscopic follow‐up based on the EAU guideline recommendations depending on their risk stratification.^1^ Patient tolerability with the procedure has been published by our group previously and so was not repeated in this study.^9^

### 2.2 Statistical analysis

Kaplan–Meier survival curves from the time of first laser ablation were produced for recurrence‐free survival, progression‐free survival, and overall survival. Five and ten‐year survival estimates were also produced with 95% confidence intervals (CI). Logistic regression analysis was carried out with NMIBC grade (low/high) as the exposure variable to calculate age‐ and sex‐adjusted odds ratios for risk of recurrence and progression; low‐grade NMIBC was used as the reference. Pearson's chi‐squared test was carried out to calculate the association between NMIBC grade and both recurrence and progression.

## 3 RESULTS

A total of 97 patients (mean age 84 [range 62–98] years) with low‐volume recurrence of NMIBC that were eligible for laser ablation between 2008 and 2016 were included. The median follow‐up time was 61 (range 2–150) months (5.1 [range 0.2–12.5] years). A total of 199 procedures were carried out on 97 patients, with a median of 1 (mean 2) procedures per patient (range 1–4). The patient group consisted of 74 (76.3%) men and 23 (23.7%) women. The breakdown of histology is shown in Table 1. The most common histology at presentation was G2Ta, seen in 45 (46.4%) patients. In total, 74 (76.3%) patients had low‐grade (G1 or low grade G2) tumors, and 17 (17.5%) patients had high‐grade (high grade G2 or G3) tumors. Histology was unavailable for six (6.2%) patients who had small tumors that appeared superficial but only underwent laser ablation without a biopsy at any point due to significant medical comorbidity and limited life expectancy precluding GA. These patients were excluded from further analysis. 39 (40.2%) patients had previously received intravesical treatment prior to laser ablation, of which 11 (28%) patients received BCG (including sequential electromotive MMC + BCG), 24 (62%) patients received MMC, and 4 (10%) patients received both BCG and MMC at separate times.

**TABLE 1:** Patient characteristics, tumor histology, and grade

Patients (*n*)	97
Procedures (*n*)	199
Age (SD)	83.56 ± 7.46
Gender	
Male	74 (76.3%)
Female	23 (23.7%)
Histology	
G1 Ta	24 (24.7%)
G2 Ta	45 (46.4%)
G2 T1	5 (5.2%)
G3 Ta/CIS	9 (9.3%)
G3 T1/CIS	8 (8.2%)
TCC N/S	6 (6.2%)
Grade	
Low‐grade (G1‐ G2)	74 (76.3%)
High‐grade (G2‐G3)	17 (17.5%)
Grade N/S	6 (6.2%)

### 3.1 Recurrence

Overall, there were 105 recurrences in 55 (56.7%) patients with a mean time from laser ablation to recurrence of 15.8 ± 12.3 months (1.31 ± 1.03 years). This equated to 1.91 ± 1.09 recurrences per patient and the number of recurrences ranged from 1 to 5. Of these, 28 (50.9%) patients had one recurrence, 10 (18.1%) had two, 12 (21.8%) had three, 4 (7.2%) had four, and 1 (1.8%) patient had five recurrences. Patients who recurred had a mean age of 83.47 ± 7.09, and 45 (82%) had low‐grade tumors at presentation (Table 2).

**TABLE 2:** Overall recurrence and progression rates based on original tumor grade and stage

Original histology	Recurrence rate (%)	Progression rate (%)
G1 Ta	15/24 (62.5%)	7/24 (29.2%)
G2 Ta	29/45 (64.4%)	1/45 (2.2%)
G2 T1	1/5 (20%)	0/5 (0%)
G3 Ta/CIS	5/9 (55.6%)	1/9 (11.1%)
G3 T1/CIS	3/8 (37.5%)	0/8 (0%)
TCC not specified	2/6 (33.3%)	0/6 (0%)
Overall	55/97 (56.7%)	9/97 (9.3%)
Grade		
Low‐grade (G1‐G2)	45/74 (60.8%)	8/74 (10.8%)
High‐grade (G2‐G3)	8/17 (47.1%)	1/17 (5.9%)
Grade not specified	2/6 (33.3%)	0/6 (0%)
Time to:	Recurrence (months)	Progression (months)
Mean	15.8	24.1
Standard Deviation	12.3	20.7

**FIGURE 1:**
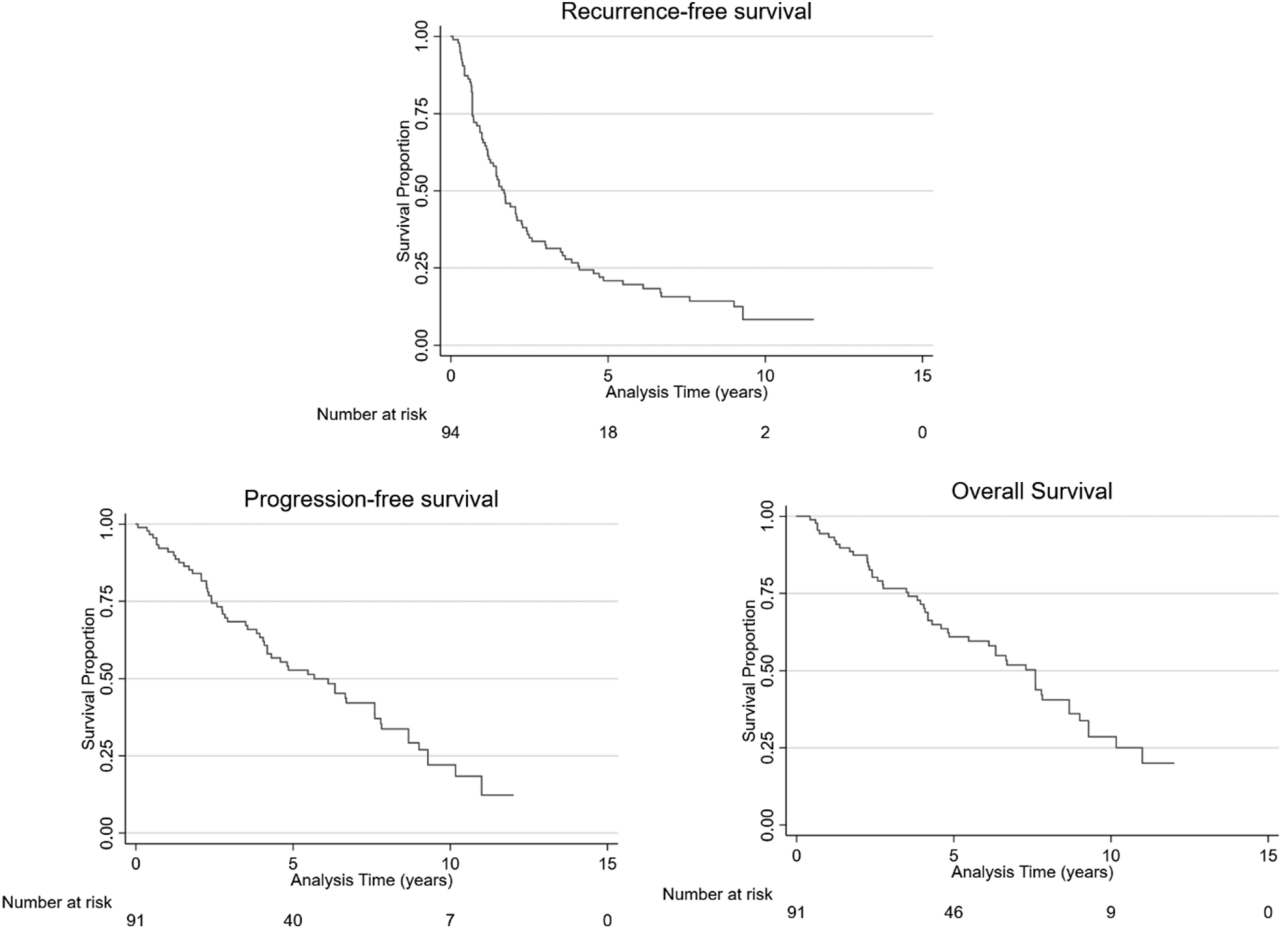
Kaplan–Meier curves for recurrence‐free, progression‐free, and overall survival estimates at 5 and 10 years

The 5 and 10‐year recurrence‐free survival rate was 21% (95% CI 13–30) and 8% (95% CI 3–17), respectively (Figure 1). The median recurrence‐free survival was 20 months (1.69 years, 95% CI 1.20–2.25) (Table 3). Age and sex‐adjusted odds ratios for risk of recurrence was calculated for high‐grade NMIBC (OR 0.51 95% CI 0.20–1.33) with low‐grade NMIBC used as the reference. Chi‐squared test indicated that there was no association between original NMIBC grade and recurrence (Pearson's chi^2^[1] = 0.3108, *p* = 0.577).

**TABLE 3:** Recurrence‐free, progression‐free and overall survival estimates at 5 and 10 years

Survival	Median survival (years) (95% CI)	5‐year estimate (%) (95% CI)	10‐year estimate (%) (95% CI)
Recurrence‐free	1.69 (1.20–2.25)	21 (13–30)	8 (3–17)
Progression‐free	5.70 (4.10–7.60)	53 (41–63)	22 (12–34)
Overall	7.60 (4.90–8.70)	61 (49–71)	29 (17–41)

### 3.2 Progression

Nine (9.3%) patients with a mean age of 82.67 ± 6.28 had tumor progression to a higher grade or stage, which included eight (88.8%) patients with initially low‐grade tumors and one (11.1%) patient with a high‐grade tumor (Table 2). With regard to histology, six (6.2%) patients with G1 Ta progressed to low‐grade G2 Ta, one (1.0%) patient with G1 Ta progressed to G3 Ta, one (1.0%) with G2 Ta progressed to G3 Ta, and one (1.0%) patient with G3 Ta progressed to G3 T1 (Table 2). Importantly, no patients progressed to muscle‐invasive or metastatic disease.

The mean time from laser ablation to progression was 24.1 ± 20.7 months (2.01 ± 1.73 years). The progression‐free survival rate for 5 and 10 years was 53% (95% CI 41–63) and 22% (95% CI 12–34), respectively (Figure 1). The median progression‐free survival was 68 months (5.70 years, 95% CI 4.10–7.60) (Table 3). Age and sex‐adjusted odds ratios for risk of progression were calculated for high‐grade NMIBC (OR 0.57 95% CI 0.07–5.24) with low‐grade NMIBC used as the reference. Chi‐squared test indicated that there was no association between original NMIBC grade and progression (Pearson's chi^2^[1] = 0.5769, *p* = 0.448).

### 3.3 Complications and mortality

The average Charlson Comorbidity Index score was 6.93 ± 1.60. No patients required emergency inpatient admission within 30 days following the laser ablation, and no patients developed clot retention or required blood transfusion. At the time of data collection, 52 (53.6%) patients in the cohort had died. The mean time from laser ablation to death for these patients was 55.5 ± 35.2 months (4.60 ± 2.93 years) (range 5.13–132.06 months).

Patients in this study had an estimated overall survival of 61% (95% CI 49–71) and 29% (95% CI 17–41) at 5 and 10 years, respectively (Figure 1). The median overall survival was 7.60 years (95% CI 4.90–8.70).

## 4 DISCUSSION

This study has demonstrated the satisfactory long‐term efficacy and safety of office‐based Holmium:YAG laser ablation for the management of recurrent low and intermediate‐risk NMIBC. The current evidence base is predominantly from small case series with short‐term follow‐up. There is, therefore, a paucity of data on long‐term outcomes, including recurrence, time to recurrence, progression, mortality, and complications, and this study provides this long‐term evidence on the safety and effectiveness of office‐based laser ablation as an alternative to the more invasive and expensive approaches of transurethral resection or fulguration under general anesthesia.

In this study, the overall tumor recurrence rate was 56.7% with a mean follow‐up time of 64.4 months (5.36 years), which is consistent with previously reported recurrence rates of up to 73% following laser ablation.^11^, ^12^ This is also similar to the 57.8% recurrence rate reported in the only other long‐term study of laser ablation at a follow‐up time of 69.8 months ablation.^11^, ^12^ The majority (76.3%) of patients in the present study had low‐grade histology, and patients with G2 Ta had the highest overall recurrence rate of 64.4%, followed by G1 Ta with a recurrence rate of 62.5%; 9.3% progressed to a higher grade or stage, but no patients progressed to muscle‐invasive or metastatic disease, and this may be related to strict patient selection for this procedure. Few studies have assessed progression rates after laser ablation for NMIBC, but where reported, the rates of progression to muscle‐invasive disease range from 6% to 9% in a population with a similar proportion of high grade or T1 tumors to the present study.^11^, ^13^

Recent recommendations from the International Bladder Cancer Group state that patients with recurrent low‐grade Ta tumors be managed with a less intensive approach than repeated transurethral resection in the operating room.^5^ Evidence from the present study demonstrates that treatment with laser ablation can achieve this aim without compromising oncological outcomes in appropriately‐selected patients. Interestingly, we found no association between grade of tumor and recurrence or progression rates, but this is likely due to the relatively small sample size of those with high‐grade tumors and those that progressed. However, this also highlights the fact that appropriate patient selection is important when offering this minimally invasive treatment, and we preferentially offer TURBT to those with high‐grade tumors unless the patient is unfit for GA or the aim of treatment is palliative. This approach of risk‐adapted management has been increasingly supported, with the aim of avoiding overtreatment of those with low‐grade Ta recurrences, but ensuring more intensive management for those with high‐grade tumors.^14^

Currently, transurethral resection of the bladder tumor (TURBT) is the gold standard procedure for recurrent NMIBC; however, this treatment is associated with side effects and complications, such as bleeding, obturator nerve reflex, and bladder perforation.^15^ In this study, no patients had complications that required inpatient admission to hospital. These data are consistent with the low number of minor complications reported in the studies by Syed et al.,^13^ including haematuria and dysuria with no major complications reported, and by Wong et al.,^9^ which reported no complications for any of the 54 patients undergoing office‐based laser ablation. Although measures such as tolerability and cost of Holmium:YAG have not been reported in this study, several previous studies have found the procedure to be well tolerated based on low visual analog pain scores and cost‐effective in different healthcare systems.^6^, ^9^, ^16^, ^17^

Furthermore, the use of this laser in an office‐based setting has the added benefit of not requiring GA. General anesthesia can have severe adverse effects on the elderly population, including postoperative delirium and cognitive dysfunction, both of which can delay rehabilitation and contribute to increased morbidity and mortality.^18^ Office‐based laser ablation is therefore a safer treatment option for elderly patients with multiple comorbidities, those on anticoagulation, or those who are unsuitable for GA. In the present study, the Charlson Comorbidity Index was used to assess the average estimated survival rate for this cohort based on pre‐existing comorbidities. This study population was elderly and comorbid, with a mean age of 83.6 and an average Charlson Comorbidity Index of 6.93. Putting this into context, the estimated 10‐year survival of a patient with a Charlson Comorbidity Index of 7 leads to a predicted 10‐year survival of 0%.^10^ Outpatient‐based laser ablation is therefore a good alternative to TURBT to reduce recurrence rates in this comorbid group, with minimal morbidity.

There are several limitations to report regarding this long‐term study. This was a retrospective study without a comparator group, and only select patients underwent treatment with laser ablation. There was heterogeneity in terms of histological subtypes included which could affect the recurrence rates reported, and we did not report outcomes based on tumor size, number, or prior treatment with intravesical therapy. Furthermore, we did not have data on site of recurrence following laser ablation which would be interesting in assessing the efficacy of laser ablation. Biopsies were not taken at the time of laser ablation, and so this may affect the outcome data in terms of upstaging. However, there is evidence that a skilled urologist can accurately identify low‐grade, non‐invasive recurrent papillary bladder tumors without the need for biopsy and can be treated with fulguration alone.^19^ We did not have data on immediate post‐procedure haematuria or UTI rates, and no qualitative data, such as patient satisfaction, tolerability, pain, and quality of life, were recorded; however, as discussed earlier, our group has previously reported these aspects.^9^ However, this study provides evidence for the long‐term efficacy and safety of this minimally invasive treatment and will prove valuable in counseling suitable patients with low‐grade recurrent NMIBC in an attempt to minimize the morbidity and cost of long‐term management.

## 5 CONCLUSION

We have shown that office‐based Holmium:YAG laser ablation is an oncologically safe method of managing recurrent low‐grade non‐muscle‐invasive bladder cancer in the long‐term, with no patients progressing to muscle‐invasive disease. Furthermore, the procedure is safe, and no significant complications were seen in this elderly and comorbid population. The procedure is optimal for those with low‐grade Ta recurrences, and patient selection is important in ensuring satisfactory oncological outcomes. The long‐term efficacy reported in this study will prove valuable in counseling patients who may be appropriate for this treatment.

## CONFLICT OF INTEREST

The authors declare no conflict of interest.
